# Beneath the Skin: Cutaneous Manifestations of Polyarteritis Nodosa With Suspected Renal Involvement

**DOI:** 10.7759/cureus.118024

**Published:** 2026-10-16

**Authors:** Hetvi A Patel, Harsh Patel, Parul Bhatt

**Affiliations:** 1 Department of General Medicine, Gujarat Medical Education and Research Society (GMERS) Medical College and Hospital Sola, Ahmedabad, IND; 2 Department of General Medicine, Dr. M.K. Shah Medical College &amp; Research Centre &amp; Smt S.M.S. Multispeciality Hospital, Ahmedabad, IND

**Keywords:** anca-negative vasculitis, cutaneous vasculitis, fibrinoid necrosis, medium-vessel vasculitis, necrotic skin ulcers, polyarteritis nodosa, pulse cyclophosphamide, renal involvement

## Abstract

Polyarteritis nodosa (PAN) is a rare systemic necrotizing vasculitis that predominantly affects medium-sized muscular arteries and is typically not associated with antineutrophil cytoplasmic antibodies (ANCA). We report the case of a 40-year-old man with a history of hypertension and a three-year history of relapsing and remitting episodes of fever. It was also associated with recurrent cutaneous lesions that temporarily improved with corticosteroid therapy received from an outside physician. He reported bilateral lower-limb tingling for which he was taking oral herbal supplements. He presented with cutaneous manifestations of necrotic ulcers, hemorrhagic bullae with peripheral erythema, and purpuric plaques. He also had associated bilateral progressive lower limb edema and bilateral joint pain. Laboratory evaluation revealed a white blood cell count of 24,900/µL and a C-reactive protein level of 140 mg/L. Serum creatinine was 1.70 mg/dL, with an estimated glomerular filtration rate of 53 mL/min/1.73 m² and blood urea nitrogen (BUN) of 28 mg/dL. Urine routine and microscopy showed +2 proteinuria without RBC casts or hematuria. Urinary microalbumin was 73.73 mg/L. Both p-ANCA and c-ANCA were negative, while hepatitis B surface antigen and anti-hepatitis C virus antibody testing were non-reactive. C3, C4, and IgA levels were within normal limits, and antinuclear antibody (ANA) was negative. Histopathological examination demonstrated cutaneous vasculitis characterized by a neutrophilic infiltrate with leukocytoclasia, perivascular distribution, focal endothelial swelling, fibrinoid necrosis, and thrombosis, predominantly involving medium-sized vessels. Direct immunofluorescence was negative for IgG, IgM, IgA, and C3, with no immune deposits in the vessel walls; together with negative ANCA and ANA testing, normal IgA and complement levels, and a negative infectious work-up, these findings helped narrow the differential diagnosis. The biopsy findings, in conjunction with the clinical presentation, supported a diagnosis of cutaneous PAN. The concurrent renal biochemical abnormalities raised concern for possible renal involvement; however, definitive renal vascular evaluation could not be performed. Although renal involvement attributable to PAN was not definitively established, it was clinically suspected by the nephrology and rheumatology teams. The patient received intravenous pulse cyclophosphamide (15 mg/kg once monthly for six months) in combination with systemic corticosteroid therapy consisting of an initial intravenous methylprednisolone dose followed by oral prednisolone (1 mg/kg/day) with gradual taper. Empirical antibiotics were administered along with topical wound care for the skin lesions. Following the initial treatment cycle, the white blood cell count decreased to 11,400/µL, the C-reactive protein level decreased to 9.60 mg/L, and the cutaneous lesions demonstrated marked re-epithelialization without the development of new lesions. After the completion of the treatment his renal function test (RFT) values improved and BUN was 19 mg/dL and creatinine decreased to 1.1 mg/dL. This case emphasizes that prominent cutaneous manifestations of PAN should prompt careful evaluation for systemic involvement, particularly when clinical or laboratory findings suggest possible systemic involvement.

## Introduction

Polyarteritis nodosa (PAN) is a systemic necrotizing vasculitis that typically affects medium-sized arteries and generally spares capillaries, venules, and glomeruli [1]. PAN is generally antineutrophil cytoplasmic antibody (ANCA)-negative and is classified separately from ANCA-associated vasculitides. A French population-based study estimated the prevalence of PAN at 30.7 cases per million adults, emphasizing the rarity of the disease [2]. Although most cases are idiopathic, secondary forms have been associated with infections, particularly hepatitis B virus (HBV) and, less frequently, hepatitis C virus, as well as hematologic malignancies such as hairy cell leukemia [1]. HBV-associated PAN is a well-established secondary form of the disease [3,4]. Its pathogenesis is thought to involve the deposition of circulating immune complexes containing viral antigens within affected arterial walls [3].

The clinical presentation of PAN varies considerably according to the distribution and severity of arterial involvement. Cutaneous PAN is generally considered a more benign form in which the disease is predominantly confined to the skin and subcutaneous tissues [1]. Its manifestations may include tender subcutaneous nodules, livedo reticularis, purpura, painful ulceration, and digital ischemia [1]. In contrast, systemic PAN may compromise arterial blood flow to multiple organs, particularly the peripheral nerves, gastrointestinal tract, kidneys, and heart, resulting in ischemia and tissue injury [1]. Pulmonary arterial involvement is generally absent, and glomerulonephritis is not a typical feature of PAN [1]. When the kidneys are affected, the disease primarily involves the renal arteries and may result in hypertension, renal ischemia or infarction, and impaired renal function [1].

The report describes a 40-year-old man who presented with cutaneous lesions, including extensive ulcers, hemorrhagic bullae, and purpuric plaques, along with bilateral knee arthralgia and bilateral pedal edema that had progressed to the knees. He reported a three-year history of recurrent febrile episodes and cutaneous manifestations with transient improvement following corticosteroid therapy, but no documented laboratory evaluation during these earlier episodes. At presentation, laboratory evaluation revealed elevated CRP, leukocytosis, negative antinuclear antibody (ANA) and ANCA along with elevated serum creatinine, urea and blood urea nitrogen (BUN) along with proteinuria, and microalbuminuria.

## Case presentation

Patient information and clinical presentation

A 40-year-old man with a three-year history of hypertension presented with progressive bilateral pedal edema, bilateral knee arthralgia, recurrent febrile episodes, and extensive cutaneous lesions. The pedal edema had initially involved the feet and subsequently progressed to the knees. He reported recurrent episodes of fever over the preceding three years, often lasting up to one week and associated with cutaneous manifestations. These episodes had been treated intermittently with oral deflazacort, with temporary improvement followed by recurrence. No laboratory or histopathological evaluation had been performed during these earlier episodes.

On examination, he had extensive polymorphic cutaneous lesions, including a large ulcer with necrotic tissue, hemorrhagic bullae, and multiple dusky purpuric patches and plaques involving the extremities as well as flank. Several tender, dusky lesions were present over the toes and fingertips. Bilateral pedal edema involved the feet, ankles, and lower legs, with reported progression toward the knees. Bilateral knee arthralgia was also present.

His medical history was significant for bilateral tingling and numbness of the lower limbs, for which he was taking oral herbal supplements. He had been diagnosed with hypertension three years earlier, around the time his cutaneous manifestations and febrile episodes began. He was taking losartan 50 mg once daily for hypertension and his blood pressure was controlled with medication. The subsequent laboratory findings raised suspicion for possible renal involvement. However, no renal function testing had been performed during the earlier period. Therefore, the exact temporal relationship between hypertension and renal dysfunction could not be established. He had no known history of other chronic systemic illnesses and denied weight loss, hemoptysis, or gross hematuria. He had no history of smoking, alcohol use, or tobacco consumption.

Physical examination findings

On initial clinical examination, the patient was febrile, with an axillary temperature ranging from 100°F to 101°F (reference range: 95 to 98.5°F). His pulse rate was 110 beats per minute (reference range: 60-100 beats per minute), blood pressure was 130/80 mmHg (reference value: <120/80 mmHg), respiratory rate was 18 breaths per minute (reference range: 12-20 breaths per minute), and body mass index was 26.2 kg/m². Cardiovascular examination revealed normal S1 and S2 without murmurs. He was conscious and oriented to time, place, and person. Neurological examination revealed tingling and numbness in the lower limbs. Peripheral pulses were palpable bilaterally in the upper and lower extremities and capillary refill was normal. Bilateral pitting pedal edema extended from the feet and ankles toward the knees. Cutaneous examination revealed widespread polymorphic lesions involving the upper and lower extremities. No livedo reticularis or palpable purpura were seen. A large cutaneous ulcer was present over the upper extremity, with mild surrounding erythema and an ulcer bed containing yellowish fibrinous slough and areas of necrotic tissue (Figure 1).

**Figure 1:**
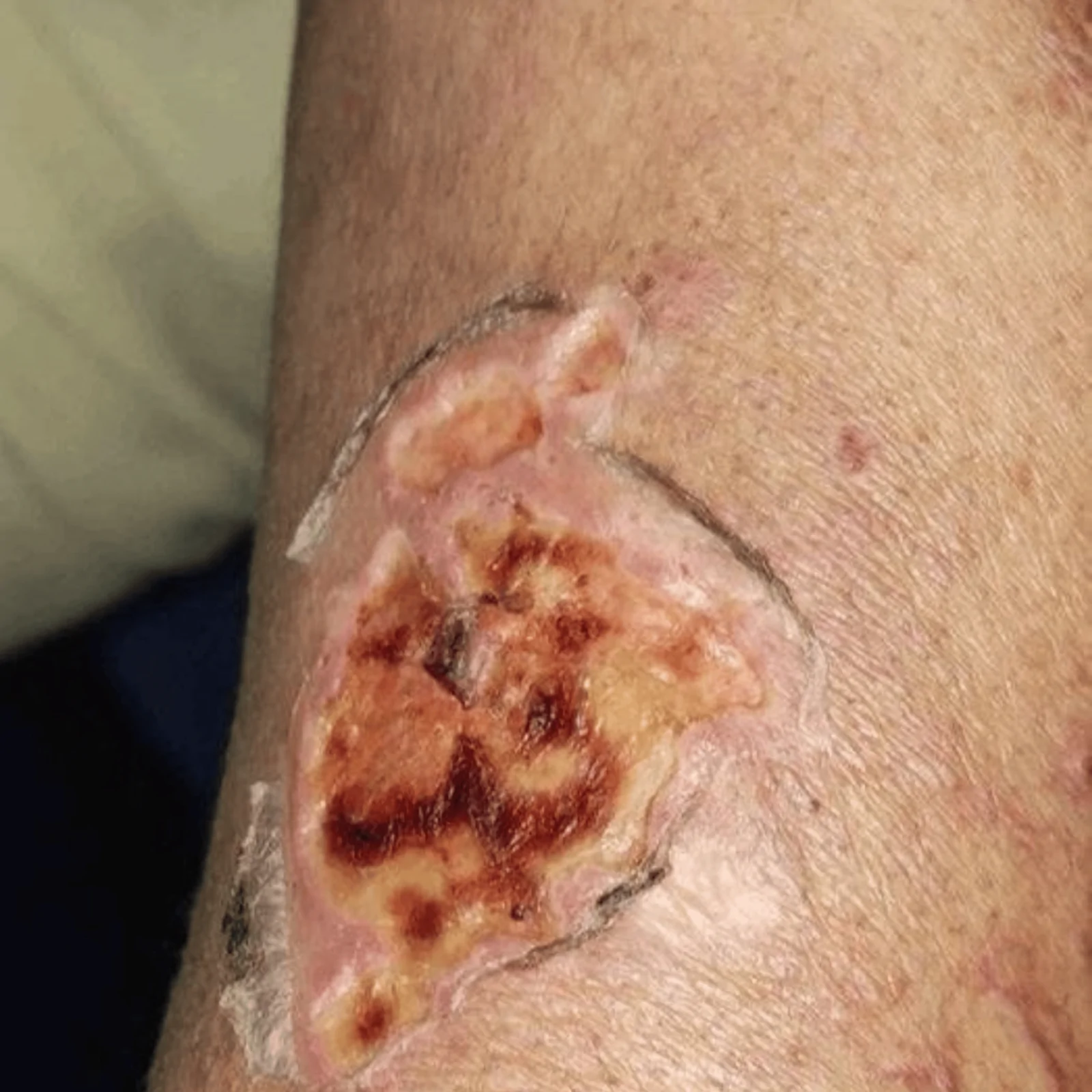
Cutaneous ulcer of the upper extremity A large cutaneous ulcer with yellowish fibrinous slough, areas of necrotic tissue within the ulcer bed, and mild surrounding erythema at the time of presentation.

Approximately 10-12 tender, dusky lesions were present over the distal toes and fingertips, several of which had a purpuric or bullous appearance. Multiple well-defined, tender, dusky purpuric patches and plaques were also distributed over the lower extremities, particularly over the dorsum of the foot and distal lower leg (Figure 2).

**Figure 2:**
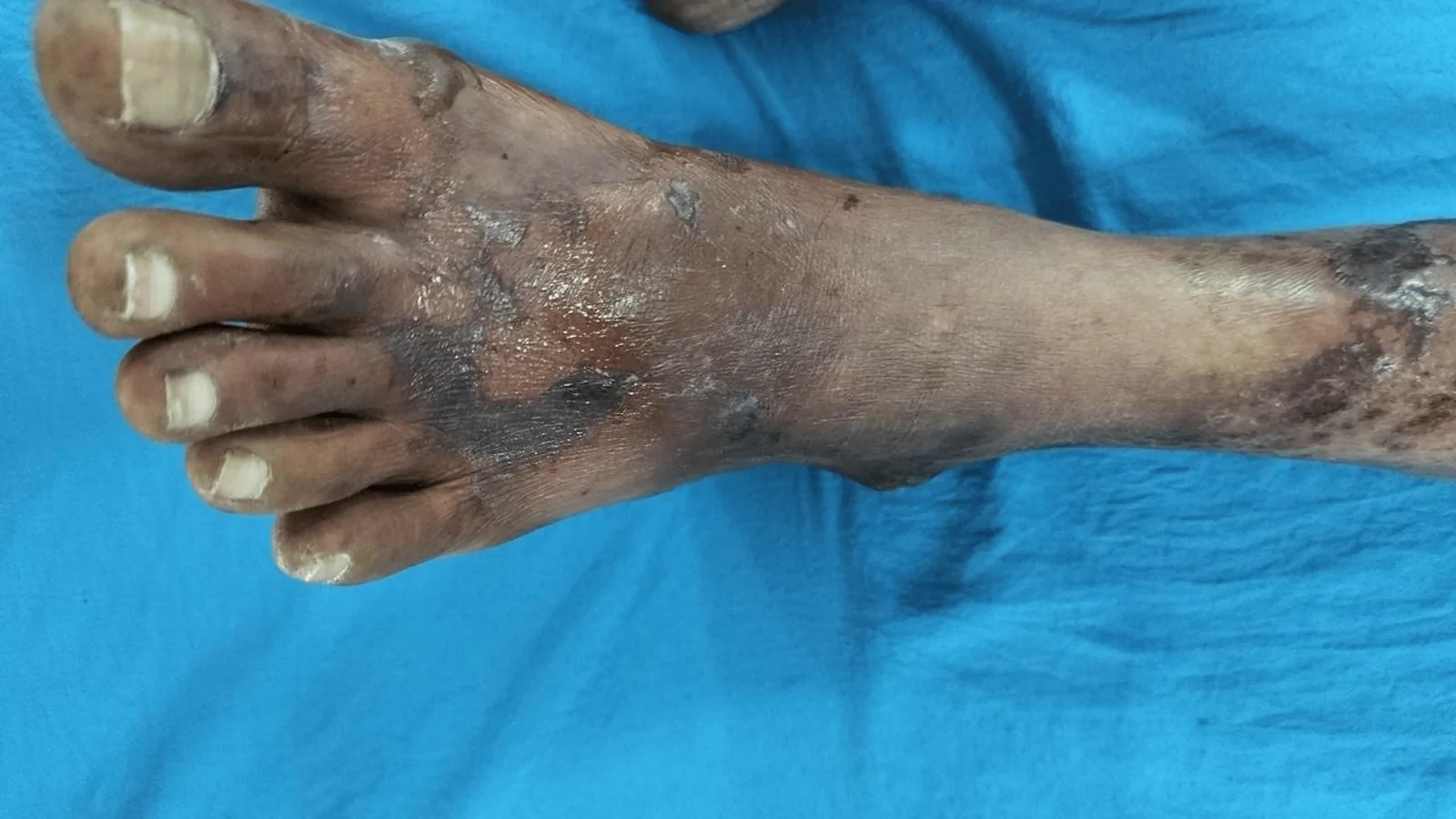
Purpuric lesions of the lower extremity Multiple well-defined, dusky purpuric patches and plaques involving the dorsum of the foot and distal lower leg at the time of presentation.

Examination of the lateral aspect of the foot demonstrated an intact fluid-filled bulla at the base of the great toe, additional dusky purpuric lesions, and painful, erythematous, pitting pedal edema involving the foot, ankle, and heel (Figure 3).

**Figure 3:**
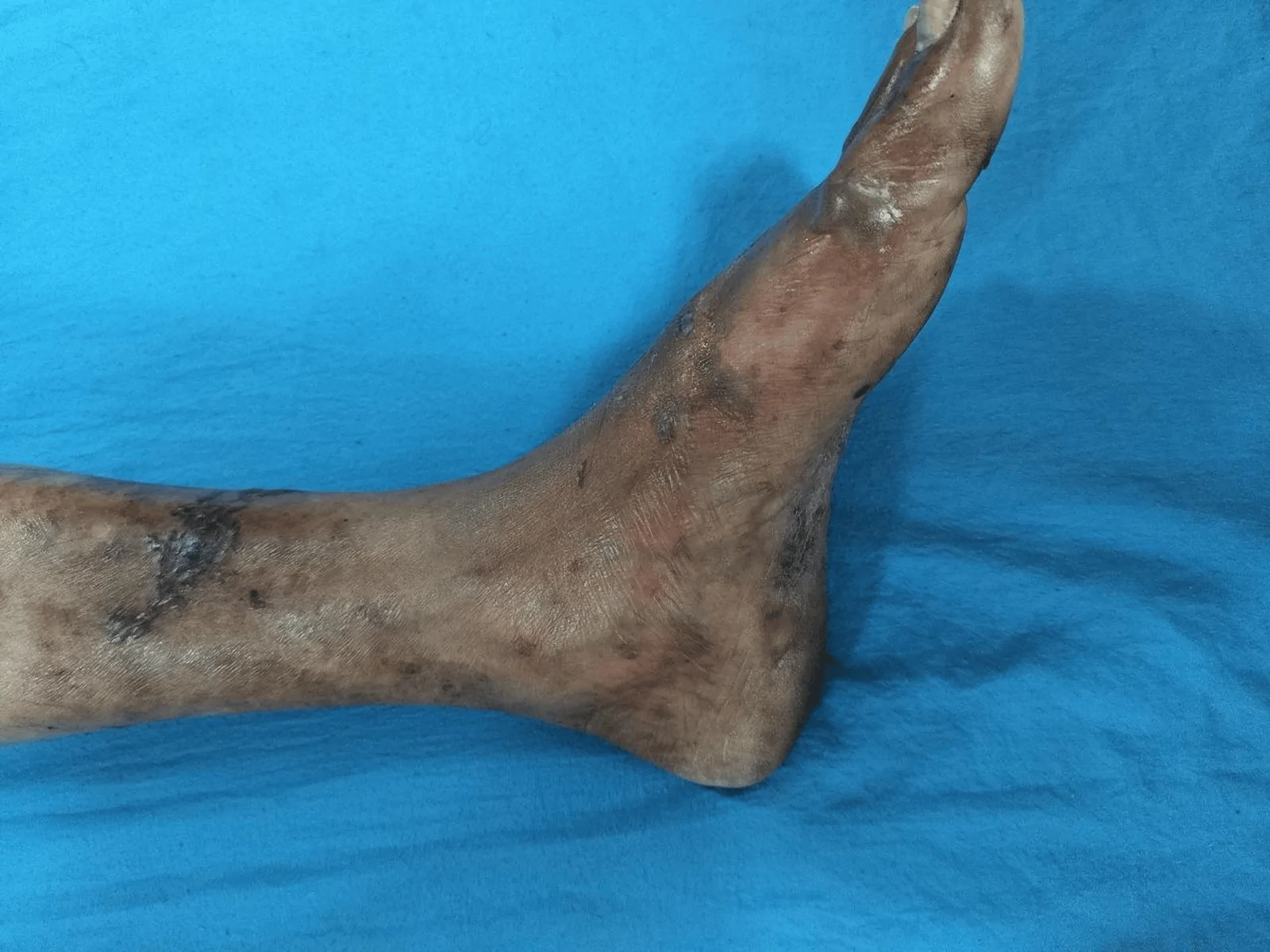
Bullous and purpuric lesions of the foot Lateral view of the foot showing an intact fluid-filled bulla at the base of the great toe, multiple dusky purpuric lesions, and pedal edema involving the foot and ankle at the time of presentation.

Laboratory and diagnostic evaluation

At the time of admission, a comprehensive laboratory and serological evaluation was performed to assess the degree of systemic inflammation and identify possible internal organ involvement. The white blood cell (WBC) count was markedly elevated at 24,900/µL, and the serum C-reactive protein (CRP) level was 140 mg/L, indicating a significant inflammatory response. Laboratory evaluation revealed a serum creatinine of 1.70 mg/dL and an estimated glomerular filtration rate of 53 mL/min/1.73 m². Urinalysis showed +2 proteinuria with no hematuria, red blood cell (RBC) casts or WBCs. Serum albumin was 3.08 g/dL, serum cholesterol was 146 mg/dL (<200 mg/dL) and total protein was 7.16 g/dL (6.5 to 8.5 gm/dL), which made nephrotic syndrome unlikely. Urine microalbumin was 73.73 mg/24 hr and urea was 57.46 mg/dl (reference range: 15-45 mg/dl) (Table 1).

**Table 1:** Laboratory and serological findings at admission

Laboratory Parameter	Patient Value	Reference Range
White Blood Cell (WBC) Count	24,900/µL	4,000–11,000/µL
Hemoglobin	12.1 g/dL	13.5–17.5 g/dL
Platelet Count	380,000/µL	150,000–450,000/µL
C-Reactive Protein (CRP)	140 mg/L	<5.0 mg/L
Serum Creatinine	1.70 mg/dL	0.60–1.20 mg/dL
Serum Urea	57.46 mg/dL	15–45 mg/dL
Estimated Glomerular Filtration Rate (eGFR)	53 mL/min/1.73 m²	>60 mL/min/1.73 m²
Urinary Microalbumin	73.73 mg/L	<30.0 mg/24 hr
Urine Protein	+2	Negative
Urine white blood cells (WBCs)	None	0–5/HPF
Red blood cell (RBC) Casts	None	None
Hepatitis B antigen (HBsAg)	Non-reactive	Non-reactive
Anti-Hepatitis C Virus (HCV) Antibodies	Non-reactive	Non-reactive
p-antineutrophil cytoplasmic antibody (ANCA)	Negative	Negative
c-ANCA	Negative	Negative
Antinuclear antibody (ANA)	Negative	Negative
Serum IgA	1.19 g/L	0.6–3.56 g/L
Complement C3	1.50 g/L	0.9–1.8 g/L
Complement C4	0.21 g/L	0.1–0.4 g/L

Ultrasonography of the abdomen and pelvis showed no significant abnormality. Renal artery Doppler ultrasonography and CT/MR angiography were not performed.

Serological screening was non-reactive for human immunodeficiency virus (HIV), hepatitis B surface antigen (HBsAg), and anti-hepatitis C virus (HCV) antibodies. Tests for dengue and chikungunya were negative, as was the rapid plasma reagin (RPR) test for syphilis. Autoimmune evaluation showed negative c-ANCA and p-ANCA by indirect immunofluorescence at a dilution of 1:20. ANA testing by indirect immunofluorescence on human larynx epidermal carcinoma-2 (HEp-2) cells was negative at a dilution of 1:80, with no identifiable staining pattern or antigen specificity. Serum IgA was 1.19 g/L (reference range: 0.6-3.56 g/L). Complement levels were within normal limits, with C3 of 1.5 g/L (reference range: 0.9-1.8 g/L) and C4 of 0.21 g/L (reference range: 0.1-0.4 g/L) (Table 1).

Biopsy and histopathological findings

A punch biopsy was obtained from an active cutaneous lesion on the patient’s flank to characterize the underlying vasculitic process. The tissue specimen measured approximately 0.6 × 0.5 × 0.5 cm. Low-power histopathological examination demonstrated epidermal orthokeratosis and a dermal inflammatory infiltrate (Figure 4).

**Figure 4:**
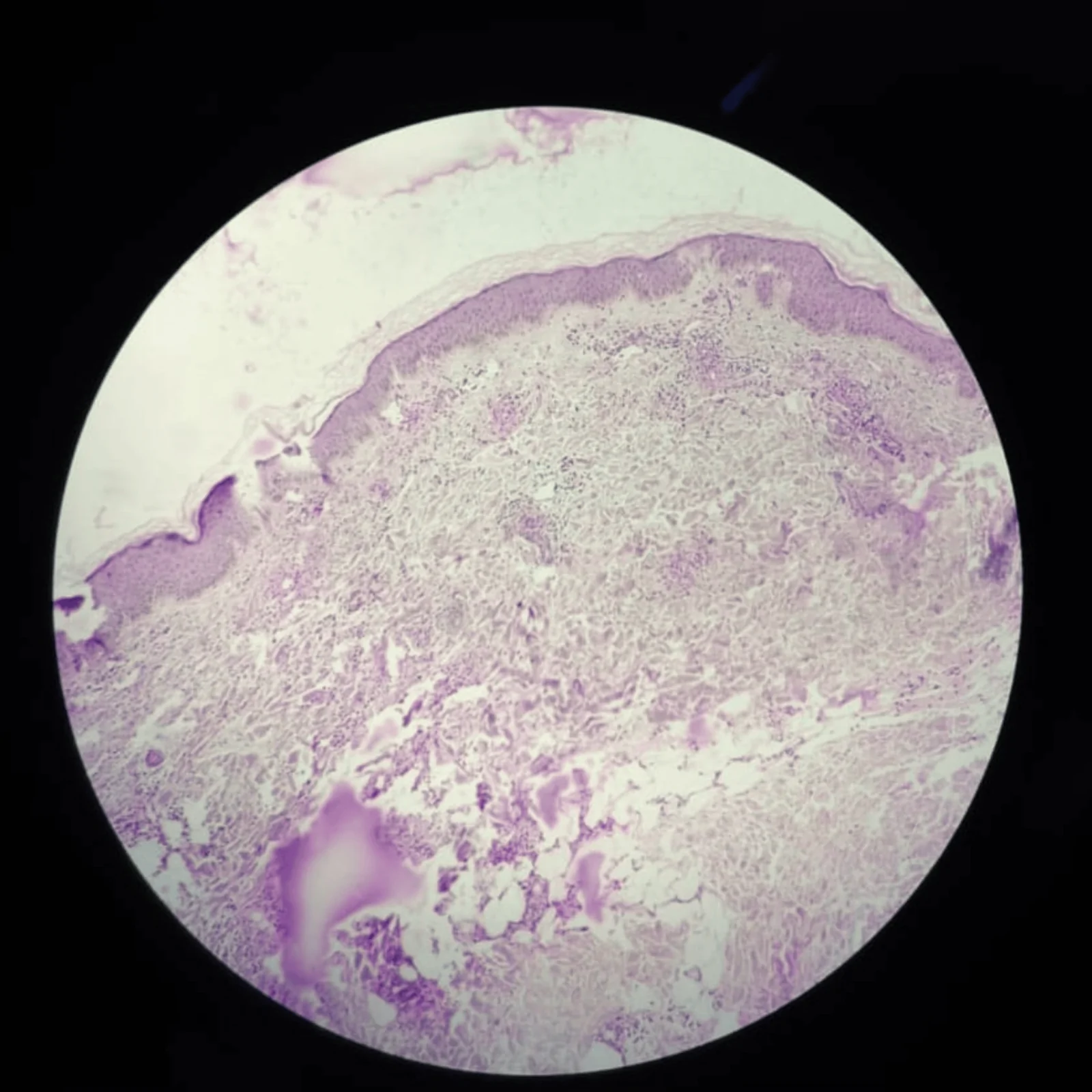
Low-power histopathological view of the skin biopsy Low-power photomicrograph showing epidermal orthokeratosis and a dermal inflammatory infiltrate (hematoxylin and eosin (H&E) stain; magnification, ×20).

High-power examination demonstrated perivascular and mural neutrophilic infiltration, focal endothelial swelling, and fibrinoid necrosis involving medium-sized blood vessels (Figure 5).

**Figure 5:**
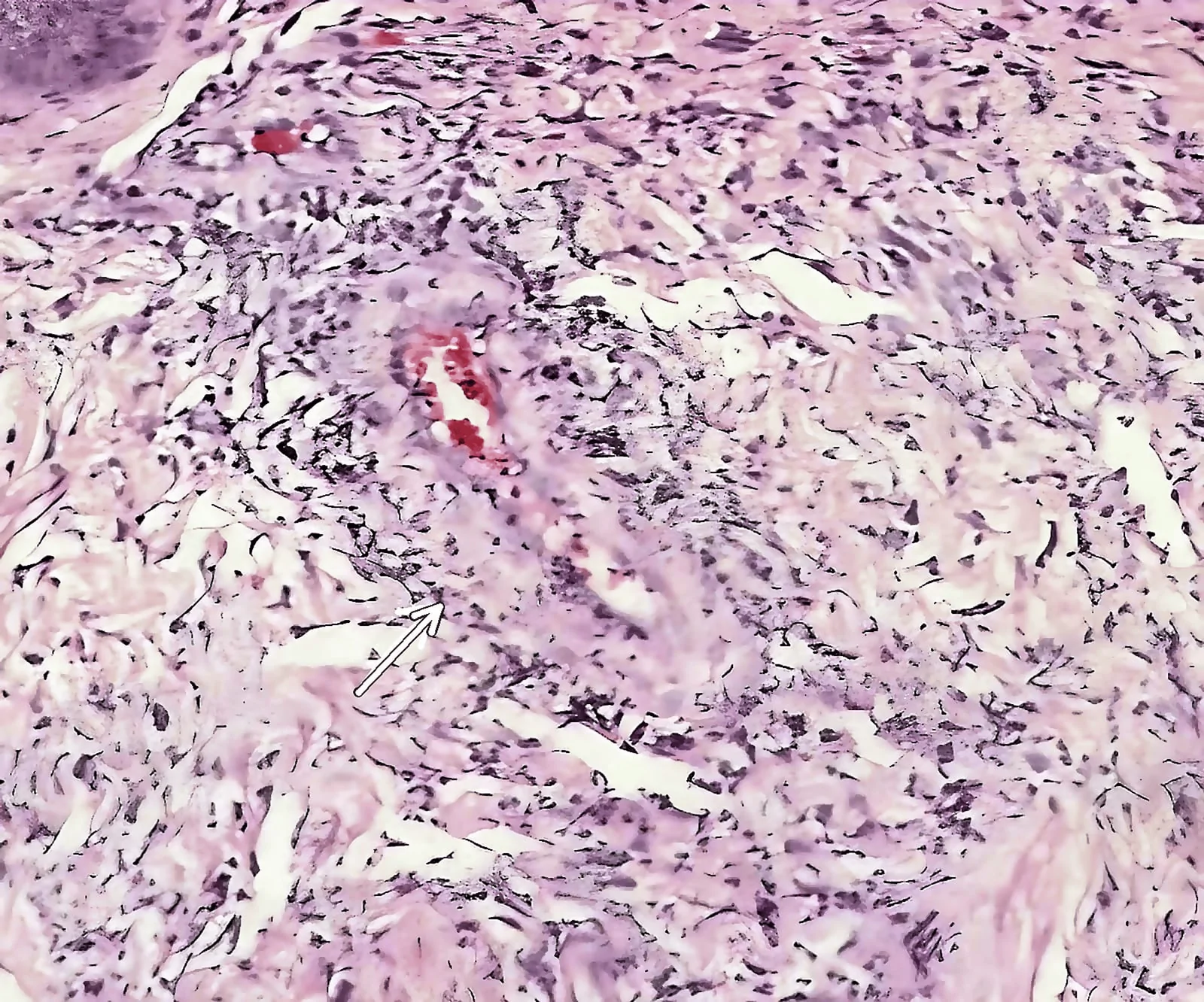
High-power histopathological view of the skin biopsy High-power photomicrograph showing perivascular and mural neutrophilic infiltration, focal endothelial swelling, and fibrinoid necrosis involving medium-sized blood vessel (hematoxylin and eosin (H&E) stain; magnification, ×40).

Additional histopathological findings included effacement of the rete ridges, occasional necrotic keratinocytes, leukocytoclasia, thrombosis, and a neutrophilic infiltrate. Granulomas were not identified. Direct immunofluorescence was negative for IgG, IgM, IgA, and C3, with no evidence of immune deposits within the vessel walls. The overall histopathological diagnosis was cutaneous vasculitis predominantly involving medium-sized blood vessels. When correlated with the clinical presentation and laboratory findings, these findings supported the final diagnosis of PAN.

Treatment and clinical outcomes

Once the diagnosis of PAN was established, intravenous pulse cyclophosphamide was initiated at 15 mg/kg once monthly, with a planned treatment duration of six months. An initial intravenous dose of methyl prednisolone 500 mg was administered for three days followed by oral prednisolone at 1 mg/kg/day for 30 days which was tapered gradually to a dose 5 mg per day by six months. As a part of pre treatment screening, Hepatitis B and C were excluded. For monitoring treatment response, WBC was checked seven days after each dose of cyclophosphamide. For infection prophylaxis cotrimoxazole was given at dose of 980 mg alternate days. Oral ascorbic acid, ferrous fumarate, calcium, vitamin D and folic acid were also provided as supportive therapy. Local wound care included topical fluticasone combined with fusidic acid, framycetin cream applied to the active ulcer beds, and calamine lotion with aloe vera applied to the affected areas. To help improve his pedal edema, furosemide 20 mg once a day injection was given for one day which was switched to oral torsemide 40 mg twice a day and was given for two more days. His pedal edema improved and then toresemide was stopped as well. He was advised to avoid fruit juice and coconut water to avoid hyperkalemia and regular monitoring was done. His serum potassium was 3.8 mEq/L (reference range: 3.5-5.1 mEq/L). 

Following the first cycle of combined immunosuppressive therapy, the patient demonstrated marked clinical and biochemical improvement. The WBC count decreased from 24,900/µL to 11,400/µL, and the serum CRP level decreased from 140 mg/L to 9.60 mg/L. After the completion of the treatment, the patient's BUN was 19 mg/dL and creatinine came down to 1.1 mg/dL. The lower-extremity cutaneous lesions showed substantial re-epithelialization, and the previously necrotic areas became dry and flattened with residual hyperpigmentation. The upper-extremity ulcer resolved clinically, leaving a flat, intact, hypopigmented scar, and no new lesions were observed (Figure 6). 

**Figure 6:**
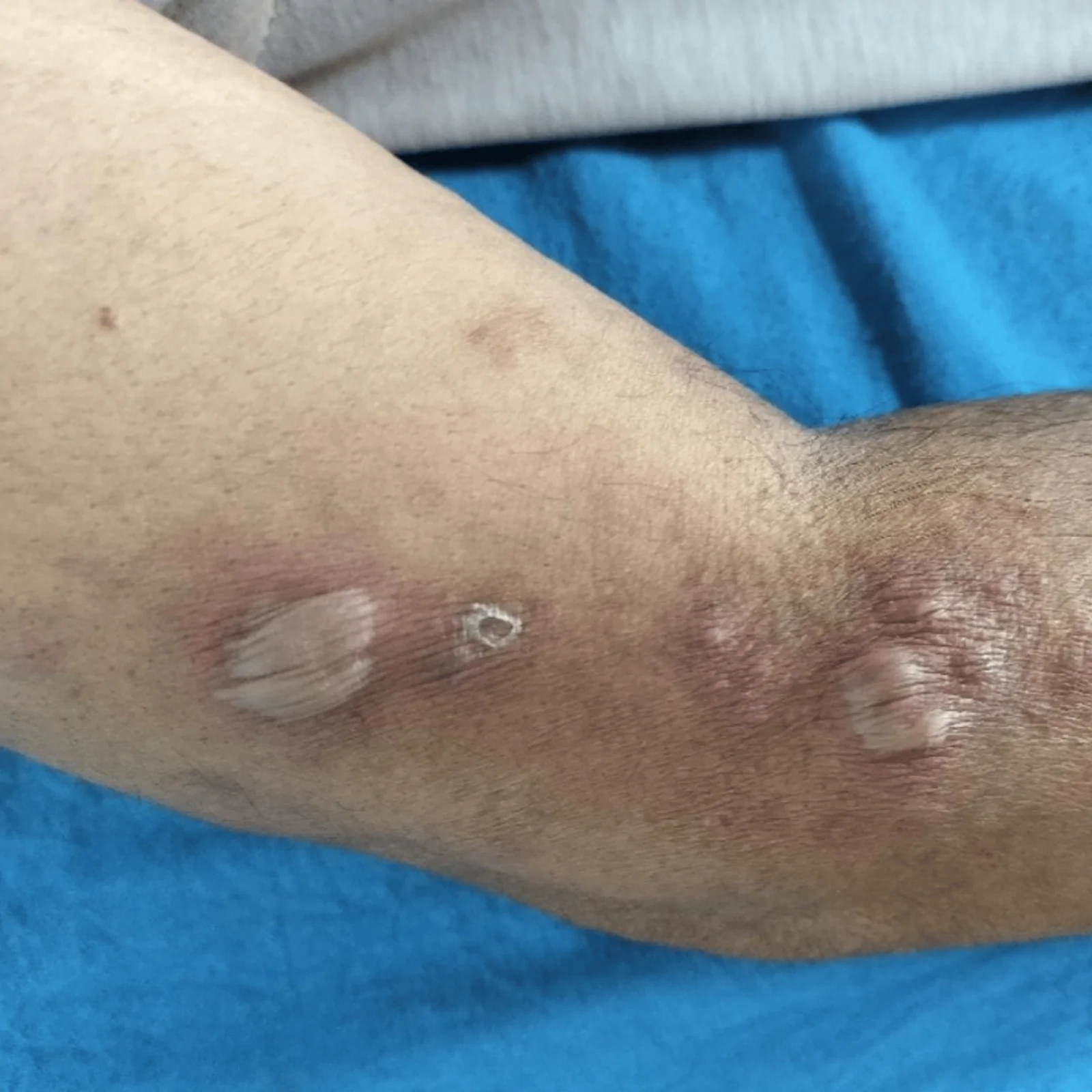
Clinical improvement of the upper-extremity lesions after the initial treatment cycle Clinical photograph obtained after the initial treatment cycle showing healing of the upper-extremity ulcer, flattening of the surrounding lesions, and residual hypopigmentation. No new lesions were observed.

## Discussion

PAN is an ANCA-negative necrotizing vasculitis that primarily affects medium-sized muscular arteries while typically sparing arterioles, capillaries, and venules [1]. Its clinical manifestations depend on the distribution and severity of vascular involvement. Cutaneous PAN is generally confined to the skin and subcutaneous tissues, whereas systemic PAN may affect the peripheral nerves, gastrointestinal tract, kidneys, and heart [1]. Distinguishing between these forms may be challenging when cutaneous manifestations dominate the presentation. In this patient, the onset of hypertension around the same time as the recurrent febrile and cutaneous episodes raised concern for possible systemic involvement. The accompanying renal biochemical and urinary abnormalities warranted further evaluation, although their cause could not be established.

Histopathology plays an important role in differentiating PAN from other vasculitic disorders. In this case, the medium-vessel necrotizing vasculitic pattern, together with the clinical presentation, supported the diagnosis of cutaneous PAN. The absence of granulomas and the negative direct immunofluorescence findings helped narrow the differential diagnosis. Negative p-ANCA and c-ANCA results were consistent with PAN and made ANCA-associated vasculitis less likely, although ANCA serology alone is insufficient to exclude these conditions. Non-reactive hepatitis B surface antigen and anti-hepatitis C virus antibody results also made active viral-associated secondary PAN less likely [1,3,4]. However, definitive renal evaluation could not be performed to determine whether the renal and urinary abnormalities were attributable to PAN, although renal involvement was clinically suspected.

Management of PAN is guided by disease severity and the extent of organ involvement [1]. Current recommendations favor intravenous pulse glucocorticoids and recommend cyclophosphamide combined with high-dose glucocorticoids for newly diagnosed active severe PAN [5]. Severe or progressive cutaneous vasculitis should prompt careful clinicopathological correlation and assessment for systemic involvement, including renal, neurological, gastrointestinal, and vascular manifestations. In this patient, combined immunosuppressive therapy was followed by substantial cutaneous healing, a reduction in inflammatory markers, and improvement in renal function.

## Conclusions

This case highlights that persistent, relapsing ulcerative, purpuric, and bullous cutaneous lesions may be a prominent manifestation of PAN and should prompt assessment for systemic disease. In this patient, renal biochemical abnormalities were identified despite the absence of overt renal symptoms, emphasizing the importance of comprehensive systemic evaluation in patients with severe or persistent cutaneous vasculitis. Careful clinicopathological correlation is essential for establishing the diagnosis and guiding immunosuppressive therapy. Further evaluation of suspected organ involvement is important to clarify the extent of disease and guide appropriate management. Early recognition and appropriate immunosuppressive treatment may reduce the risk of progressive vascular injury and irreversible organ damage.
